# C-755-08. An Oral History Archive Honoring the Legacy of Burn Nursing

**DOI:** 10.1093/jbcr/irag033.044

**Published:** 2026-04-06

**Authors:** Ashley E Buchholz, Mario Rivera, Jeffrey E Carter, M Victoria P Miles, Jonathan E Schoen

**Affiliations:** The Burn Center at University Medical Center New Orleans, Louisiana; The Burn Center at University Medical Center New Orleans, Louisiana; University Medical Center Burn Center, Louisiana State University Health Sciences Center New Orleans, Louisiana; University Medical Center New Orleans, Louisiana State University Health Sciences Center, New Orleans, Louisiana; UCI Health Regional Burn Center, New Orleans, Louisiana

## Abstract

**Introduction:**

Burn nursing was forged at the intersection of highly specialized care for the suffering and unwavering compassion. The American Nurses Association’s (ANA) recognition of burn nursing as a specialty and the creation of the Certified Burn Registered Nurse (CBRN) credential mark the culmination of a decades long journey. This project preserves that history by capturing the voices of pioneers who transformed burn nursing from an isolated practice into a recognized, evidence-based specialty.

**Methods:**

We conducted a qualitative oral history study using 10 standardized questions from veteran burn nurses and educators. Candidates were nominated by ANA and ABA officers, board members, and nursing peers. Data was collected by a CBRN at an ABA-verified burn center using in-depth remote interviews. Interview sessions explored sources of inspiration, innovations, and landmark advancements in the profession. Transcripts were thematically analyzed to trace the specialty’s chronological and philosophical evolution.

**Results:**

Data from 16 burn nursing professionals with an average of 32.6 years of experience revealed a dramatic evolution of practice grounded in humanism. Themes included three eras: Early (1960s-1980s), nurses described working in the dark ages, guided by physician-led models and limited analgesia. Dressing changes with silver sulfadiazine often meant moral distress but forged deep nurse/patient bonds. Nurses advocated within the ABA, drawing on personal survivor experiences, bringing profound empathy. Modernization (1990s-2000s) shifted care to interprofessional collaboration, nurse-driven protocols, especially for fluid resuscitation and analgesia, acknowledging nursing expertise. Early excision and dermal substitutes lessened procedural pain, and improved survival led to focus on aftercare, with nurses pioneering programs like SOAR and burn camps to help survivors thrive. In the current era (2010s-present), nurses gained formal recognition of burn nursing in 2020 from ANA and CBRN in more recent years. Mentorship emerged as essential for passing on wisdom and preserving the specialty’s ethos.

**Conclusions:**

This oral history highlights the remarkable journey of burn nursing, emphasizing its core philosophy of humanistic, patient, and family centered care. Every innovation arose from compassion and the drive to improve prevention, survival, and quality of life. Preserving these professional perspectives anchors future practice in the origin and beauty of burn nursing and the shared mission across generations.

**Applicability of Research to Practice:**

This history contextualizes best practices, reinforcing the rationale for protocols and the value of interprofessional teamwork. It illuminates mentorship as a driver of growth and leadership, inspiring nurses by connecting them to the resilience and advocacy of their predecessors.

**Funding for the study:**

Foundation Funding.

